# Epigenetic regulation of stem cells in lung cancer oncogenesis and therapy resistance

**DOI:** 10.3389/fgene.2023.1120815

**Published:** 2023-04-18

**Authors:** Jiayang Wu, Jiaming Feng, Qiran Zhang, Yazhou He, Chuan Xu, Chengdi Wang, Weimin Li

**Affiliations:** ^1^ Department of Pulmonary and Critical Care Medicine, Med-X Center for Manufacturing, Center of Precision Medicine, Precision Medicine Key Laboratory of Sichuan Province, Frontiers Science Center for Disease-Related Molecular Network, West China Hospital, West China School of Medicine, Sichuan University, Chengdu, China; ^2^ West China School of Medicine, Sichuan University, Chengdu, China; ^3^ Department of oncology, West China School of Public Health and West China Fourth Hospital, Sichuan University, Chengdu, China; ^4^ Department of Oncology, Sichuan Academy of Medical Sciences, Sichuan Provincial People's Hospital, University of Electronic Science and Technology of China, Chengdu, China

**Keywords:** epigenetics, stem cells, lung cancer, tumor microenvironment, oncogenesis, therapy resistance

## Abstract

Epigenetics plays an important role in regulating stem cell signaling, as well as in the oncogenesis of lung cancer and therapeutic resistance. Determining how to employ these regulatory mechanisms to treat cancer is an intriguing medical challenge. Lung cancer is caused by signals that cause aberrant differentiation of stem cells or progenitor cells. The different pathological subtypes of lung cancer are determined by the cells of origin. Additionally, emerging studies have demonstrated that the occurrence of cancer treatment resistance is connected to the hijacking of normal stem cell capability by lung cancer stem cells, especially in the processes of drug transport, DNA damage repair, and niche protection. In this review, we summarize the principles of the epigenetic regulation of stem cell signaling in relation to the emergence of lung cancer and resistance to therapy. Furthermore, several investigations have shown that the tumor immune microenvironment in lung cancer affects these regulatory pathways. And ongoing experiments on epigenetics-related therapeutic strategies provide new insight for the treatment of lung cancer in the future.

## 1 Introduction

Lung cancer is the most prevalent and deadly cancer in the world. Surgery, chemotherapy, radiation therapy, targeted therapy, and immunotherapy, and targeted therapy are the primary treatments for lung cancer (Hirsch et al., 2017). Despite some success with these treatments in the clinic, a significant number of patients with lung cancer still can not benefit from these therapies.

Cancer is caused by the proliferation and differentiation of aberrant cells (Hanahan and Weinberg, 2000). Therefore, the signals that could drive cell proliferation and differentiation during cancer development and progression are of particular importance in the study of cancer.

The fundamental conditions for the formation of cancer are provided by stem cells, which are somatic cells with the capacity for self-renewal and differentiation (Soteriou and Fuchs, 2018). Cells that keep stemness characteristics even after lung carcinogenesis are known as cancer stem cells (CSCs) (Batlle and Clevers, 2017). These cells play a significant role in the spread of cancer and resistance to therapy (Yang et al., 2020). Stem cell differentiation is regulated by driving differentiation signals called stem cell signals (Huang et al., 2020). It follows that it is easy to understand the significant regulatory role of this driver signal in the onset and progression of cancer.

The entire tumor system is composed of the tumor microenvironment (TME) and cancer cells (Zhang et al., 2022). The tumor microenvironment contains a significant number of immune cells, cancer stem cells respond differently to signals as prompted by their unique microenvironment (Mao et al., 2021). Therefore, a new approach for treating lung cancer in the future may depend on the understanding of these regulatory processes. Here, we focused on how epigenetic inheritance modulates these stem cell signals in the immune microenvironment, leading to lung cancer development and treatment resistance.

## 2 The origin of lung cancer and relative aging

**FIGURE 1:**
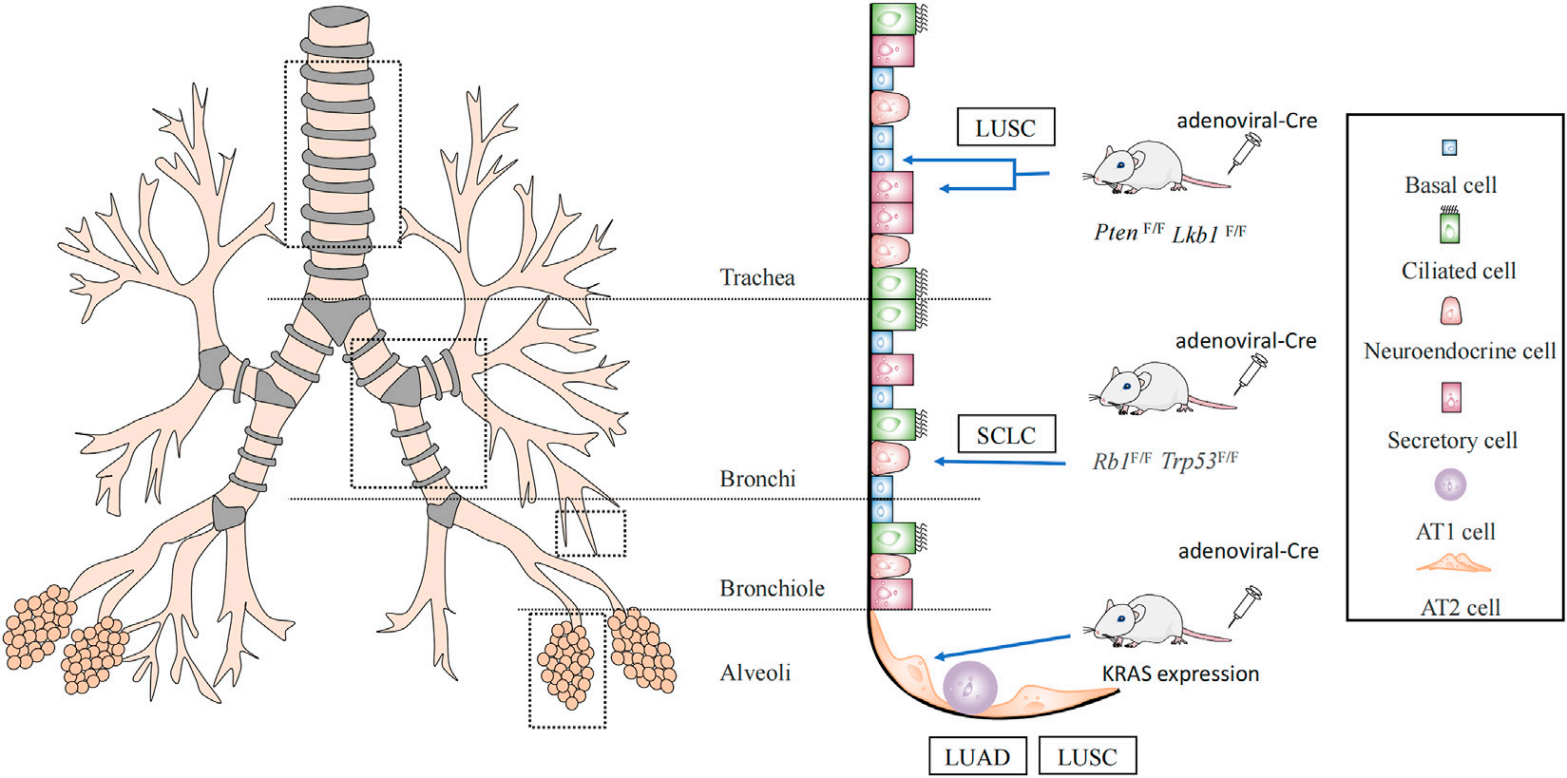
Lung cancer cells of origin. It was demonstrated by Cre mice that lung squamous cancer (LUSC) originates from basal cells with secretory cells, AT2 cells, while AT2 cells may also evolve into lung adenocarcinoma (LUAD). And small-cell lung cancer (SCLC) originates from neuroendocrine cells.

Subpopulations of cells with undifferentiated or hypodifferentiated characteristics are likely to become cancerous. Both stem and progenitor cells could be employed as the original cells for lung cancer when risk factors for lung cancer are present (Sanchez-Danes and Blanpain, 2018). Cells that initiate cancer vary depending on the pathology. Lung cancer is usually divided into small-cell lung cancer (SCLC) and non-small cell lung cancer, based on the histology (Thai et al., 2021). Approximately 85% of cases are non-small cell carcinomas, the prevalent subtypes being adenocarcinomas (LUAD), and squamous cell carcinomas (LUSC). LUAD typically develops from peripherally arising bronchioles, bronchioles, and alveoli, whereas LUSC and SCLC frequently develop from the centrally originating proximal segment bronchus to the main bronchus (Herbst et al., 2008; Ernani et al., 2022) (Figure 1).

Using a Cre mouse model, it was discovered that non-squamous epithelial basal, secretory, and alveolar epithelial type Ⅱ cell (AT2) cells are the origin of LUSC (Ferone et al., 2016). One of the most frequent genetic changes in lung LUSC is *SOX2*. Deletion of *SOX2* in human lung epithelial cells has been reported to reduce their ability to self-renew and repair tissue injury (Bass et al., 2009). Therefore, these key genes are crucial for preserving the phylogeny and stability of mature airways. *SOX2* overexpression and deletion of the tumor suppressor genes, *PTEN*, *CDKN2A*, and/or *CDKN2B* resulted in the conversion of lung basal cells, secretory cells, and AT2 cells into lung squamous cells (Ferone et al., 2016; Sanchez-Danes and Blanpain, 2018). The dominant negative of the *Maml1* mutation in secretory and AT2 cells, accompanied by *Kras* ^*G12D*^ expression and NOTCH inhibition, also causes *SOX2* expression and alveolar proliferative lesions containing squamous markers (Xu et al., 2014). In addition, the activation of *PIK3CA*, *P63*, and deletion of *LKB1* are mutations associated with squamous cell carcinoma (McCaughan et al., 2010; Mukhopadhyay et al., 2014) (Figure 1).

It has been demonstrated that AT2 cells serve as the main origin of LUAD, by using a KRAS-driven mouse model of lung cancer (Xu et al., 2012; Mainardi et al., 2014). The oncogenes, *EGFR*, *KRAS*, *BRAF*, and *PIK3CA* are frequently mutated in LUAD (Swanton and Govindan, 2016). LUAD can be triggered directly by independent *Kras* ^*G12D*^ expression. A limited number of LUAD could develop when *SOX2* expression in combination with deletion of *Trp53* or dual deletion of *Trp53* and *Rb1* in a mouse model that was constructed by the lentiviral approach. In mouse models constructed using a lentiviral approach, *SOX2* expression and deletion of *Trp53* or dual deletion of *Trp53* and *Rb1* could lead to a small number of mouses with LUAD (Sanchez-Danes and Blanpain, 2018).

Inactivating mutations in the tumor suppressor genes *TP53* and *RB* transcriptional corepressor 1 (*RB1*) are common in SCLC (Korsen et al., 2022). By conditional knockout of *TP53* and *RB1*, it was confirmed that SCLC originates mainly from neuroendocrine cells (Sutherland et al., 2011) (Figure 1). These studies suggest that the cells of origin allow for specific types of lung carcinogenesis by selecting the expression of particular genes.

## 3 Epigenetic regulation of stem cells in lung cancer oncogenesis

In addition to lung carcinogenesis being directly guided by genetic alterations, epigenetics could also control lung carcinogenesis by controlling the ability to initiate tumors. The patterns of epigenetic changes during cellular senescence and tumorigenesis are similar, with the major abnormalities being DNA and histone methylation (Xie et al., 2018; Yu et al., 2018). However, cellular senescence and tumorigenesis involve different pathways. When CPG islands are hypermethylated, they mostly affect genes that control cell synthesis and metabolism in senescent cells, and genes that control the growth and differentiation of malignant cells (Xie et al., 2018). According to this phenomenon, the epigenetic landscape is crucial because of its regulatory role in stem cell differentiation (Teschendorff and Feinberg, 2021).

Mutations in chromatin-modifying enzymes that lead to methylation modifications in DNA and histones are common in lung cancer. For example, lung cancer stem cells carry loss-of-function mutations in nuclear receptor-binding SET domain protein 1 (NSD1), which is capable of methylation-modifying histones (Garraway and Lander, 2013; Cancer Genome Atlas Research Network, 2014).

DNA methylation, histone modifications, and non-coding RNA alterations could activate multiple stem cell signals, including WNT, NOTCH, and Hedgehog, to regulate lung cancer oncogenesis and progression (Lytle et al., 2018).

This review describes how epigenetic mechanisms regulate the activation and expression of stem cell signaling pathways leading to lung carcinogenesis, mostly from two time periods: before and after gene transcription.

### 3.1 Pre-transcriptional regulation

DNA methylation was one of the first epigenetic modifications to be identified even before the establishment of the double-helix structure of DNA. DNA methylation is the process by which DNA methyltransferase (DNMT) adds a methyl group to the cytosine 5th carbon atom in the DNA sequence to create 5-methylcytosine (5 mC) (Liu et al., 2022a). Most of these alterations occur in CpG islands (CGIs). The three methyltransferases linked to methylation in humans are DNMT1, DNMT3A, and DNMT3B. After DNA replication is complete, DNMT1 methylates hemimethylated DNA during the cell division cycle, whereas DNMT3A and DNMT3B are responsible for establishing fresh DNA methylation (He et al., 2011).

The downregulation of *P53* and *P21* expression through the IL-6/JAK2/STAT pathway could upregulate DNMT1, enhance tumor initiation, and promote lung cancer stem cell proliferation (Liu et al., 2015). This demonstrates that DNA hypermethylation is associated with the silencing of oncogenes and differentiation genes in lung cancer, and that the low expression of these genes may contribute to the formation of lung cancer stem cells.

Activation of the WNT signaling pathway has been shown to be associated with increased tumor initiation potential in mouse models (Nusse and Clevers, 2017). APC, LKB1, WNT inhibitor 1 (WNT-1), Disabled-2 (Dab2), secreted frizzled-related proteins (sFRPs), and members of the Dickkopf (Dkk) family are hypermethylated silencing factors that are involved in the abnormal activations of the Wnt/β-Catenin pathway, and these aberrant activations may lead to the development of LUAD (Duruisseaux and Esteller, 2018). Upregulated G9a may silence the expression of WNT-1 through DNA hypermethylation, leading to an abnormal WNT pathway, thus affecting the growth of lung cancer cells (Zhang et al., 2018). DKK1 is a secretory protein that negatively regulates WNT signaling. The DNA hypermethylation of the DKK1 promoter promotes lung cancer growth through the WNT signaling pathway (Park et al., 2012; Han et al., 2017).

NOTCH signaling is a highly conserved intercellular communication pathway that performs multiple functions during lung development (Siebel and Lendahl, 2017), including the regulation of cell differentiation, survival, and genealogical specification. Although normal NOTCH signaling is required to maintain homeostasis *in vivo*, its abnormal activity has been shown to be associated with the development and progression of lung cancer (Allen et al., 2011; Lim et al., 2017). ASCL1 hypomethylation is common in SCLC. As a direct target of ASCL1, DLL3 (a NOTCH inhibitor) was significantly correlated with its expression status (Sabari et al., 2017).

The Hedgehog (Hh) pathway is an evolutionarily conserved signaling axis that is essential for regulating a variety of fundamental biological processes. The Hh ligands, repair receptor (PTCH), smooth intermediator (SMO), and zinc finger-containing glioblastoma transcription factor (GLI) are the four main elements of the Hh pathway. In the absence of PTCH inhibition, SMO activates the Gli transcription factor, thereby activating cancer-related target genes (Kasiri et al., 2017). The human contains Gli1, Gli2, and Gli3 proteins. Gli2 and Gli3 primarily function as Shh-regulated transcriptional activators and repressors, respectively, whereas Gli1 primarily functions as a transcriptional activator and amplifies Shh signals in a positive feedback loop (Fu et al., 2016). A regulatory pathway consisting of the ERK/PIK3/Hedgehog signaling pathway is affected by aberrant DNA methylation of 256 negatively associated genes. This signaling pathway regulates cell death and survival, and is implicated in squamous cell tumorigenesis (Shi et al., 2017). Set7-mediated methylation of Gli3 at the K436 and K595 sites increases the stability of Gli3 and its ability to bind to DNA, thereby activating Shh signaling and contributing to the development of non-small cell lung cancer (Fu et al., 2016).

Histones could be modified in a diverse range of ways, including acetylation and methylation. The two major roles of covalent histone modifications are to silence the expression of specific genes and promote transcription. Specific histone lysine methyltransferases could methylate K4, K9, K27, K36, and K7 sites in histone H3 and K20 sites in histone H4 respectively (Wang et al., 2018b). While methylation of H3K9, H3K27, and H4K20 inhibits gene transcription, methylation of H3K4, H3K79, and H3K36 increases it (Guo et al., 2019). Histone acetylation leads to a decrease in the affinity between histones and DNA, thereby facilitating transcription. In contrast, histone deacetylation removes acetyl groups and inhibits transcription through HDACs (Montgomery and Srinivasan, 2019). The methylation of H3 histones, such as H3K79me2, was found to decrease the expression of several WNT repressors to increase WNT signaling in research on the epigenetic modification of H3 histones by triptolide in NSCLC (Liang et al., 2019). In addition to changing the level of H3K4 methylation and regulating the NOTCH pathway, KDM5A, a demethylase of H3K4, also exhibits reciprocal epistasis with NOTCH 2 in ASCL1 and neuroendocrine differentiation. This further demonstrates the significance of H3K4 methylation in SCLC formation (Oser et al., 2019). HDACs could affect lung carcinogenesis and progression in the Hh pathway by changing the acetylation status of histones in the promoter region. HDAC could interact with GLI1, causing *SOX2* promoter activity to be expressed (Wei et al., 2021) (Table 1).

**TABLE 1:** Epigenetic targets acting on lung carcinogenesis and their downstream stem cell-related signaling pathways or targets.

Type	Epigenetic regulatory targets	Downstream Pathway/Targets
DNA methylation	DKK1 promoter	WNT
	ASCL1	DLL3
	WNT -1	WNT
Histone modification	H3K79me2	WNT -1
	H3K4	NOTCH/NOTCH 2
Non-coding RNA	JMJD6	WNT/β-catenin
	DNMT3A	WNT/β-cateni
	Fbxw7	NOTCH 1
	GLI2	Hedgehog
	PTCH 1	Hedgehog
	NOTCH 1	NOTCH
	SPOP	Hedgehog
	CTNNB1	WNT/β-catenin
	β-catenin	WNT

In addition, pre-transcriptional DNA methylation with histone modifications may increase the sensitivity of cells to transformation. Therefore, understanding the mechanisms of the pre-transcriptional epigenetic regulation of stem cells is an important strategy for controlling the development of lung cancer.

### 3.2 Post-transcriptional regulation

Non-coding RNAs play an essential role in the pathology of cancer. The role of long non-coding RNAs (LncRNAs) is currently poorly understood, while microRNAs (miRNAs) are imbalance regulated non-coding RNA isoforms that have received the most research attention in lung cancer.

MiR-708-5p directly inhibits the translation of DNMT3A, leading to hypomethylation in A549 and Calu-3 cells and an increase in the expression of the tumor suppressor CDH1. This reduces the activity of the Wnt/β-catenin signaling pathway and affects the development of NSCLC by altering stem cell characteristics (Liu et al., 2018). miR-770 activates the WNT/β-catenin pathway by directly binding to the 3′-UTR of JMJD6 mRNA and downregulating JMJD6 expression which, leading to non-small cell carcinogenesis (Zhang et al., 2017). miR-27a plays an oncogenic role in human lung tumorigenesis. Fbxw7, which is inhibited by the overexpression of miR-27a, could regulate cell cycle progression, including c-Myc, c-Jun, cyclin E1, and NOTCH 1 (Wang et al., 2011a). Lung cancer considerably expresses the Hh pathway in comparison to nearby normal tissues. The miR-182-5p/GLI2 axis controls lung adenocarcinogenesis by influencing the Hh pathway (Seidl et al., 2020). miR-212 causes lung carcinogenesis by directly targeting the Hh pathway receptor, PTCH1, resulting in the inhibition of PTCH1 (Li et al., 2012). The expression of miR-520b was significantly upregulated in NSCLC samples compared with normal samples. Additionally, miR-520b was found to promote NSCLC tumorigenesis through the SPOP-GLI2/3 axis (Liu et al., 2019).

The Long non-coding RNA small nucleolar RNA host gene 11 (SNHG11) could promote lung carcinogenesis through two distinct WNT pathways. The first pathway activates the WNT/β-catenin pathway *via* the SNHG11/miR-4436a/CTNNB1 ceRNA axis. In the second pathway, SNHG11 directly binds to *β*-catenin and activates the WNT pathway (Liu et al., 2020). A novel long intergenic non-protein coding RNA (LINC01783) that suppresses miR-432-5p, a route that results in non-small cell carcinogenesis, activates the NOTCH pathway to increase DLL-1 expression and enhances the proliferation of NSCLC cells (Deng et al., 2021) (Table 1).

The gain and loss of epigenetic modifications at all stages of transcription may lead to the development of lung cancer. Lung cancer stem cells are known to be preferentially affected by this process. Therefore, the regulation of lung cancer stem cells using these modification pathways as targets would be very effective therapeutic strategy for lung cancer.

## 4 Stem cell states in therapy resistance

Therapy resistance is a challenge in the clinical treatment of lung cancer. Existing treatment regimens typically fail to eliminate all tumor cells, and residual cells are believed to be the key driver of cancer recurrence in patients (Lytle et al., 2018).

### 4.1 Therapy resistance mechanisms involved in lung cancer stem cells

Lung cancer stem cells participate in drug resistance by hijacking the properties of normal stem cells. The three most common pathways are drug transport, DNA damage repair, and niche protection (Hovinga et al., 2010; Blanpain et al., 2011; Assaraf et al., 2019).

#### 4.1.1 Drug transport

**FIGURE 2:**
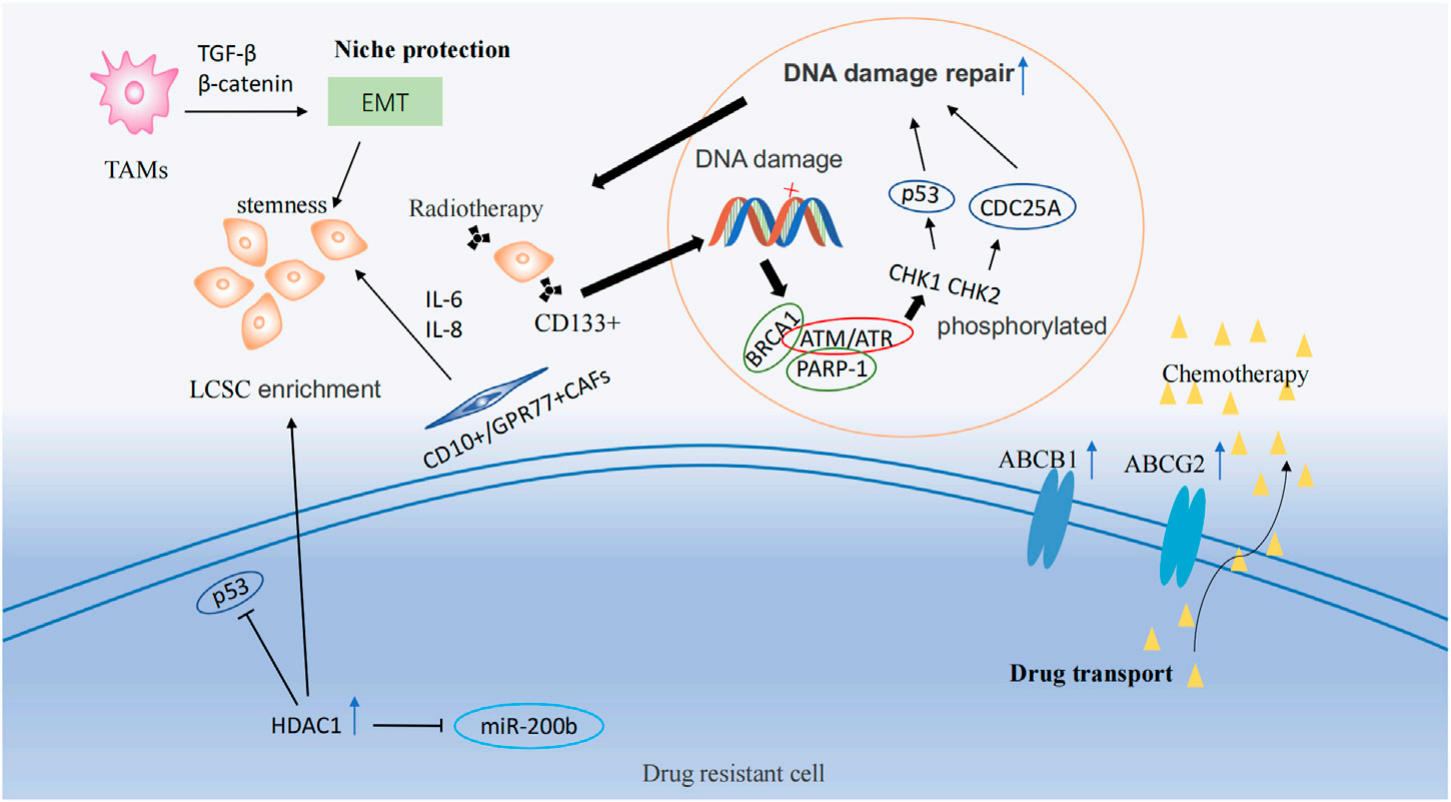
Drug resistance mechanisms and epigenetic regulation involving tumor stem cells. Drug resistance mechanisms include drug transport, DNA damage repair, and ecological niche protection processes. LSCS: lung cancer stem cell; TAMs: Tumor-associated macrophages; EMT: Epithelial-Mesenchymal Transition; CAFs: cancer associated fibroblasts.

ATP-binding cassette (ABC) transport proteins are the main proteins that regulate the efflux of cytotoxic drugs (Greenwood et al., 2019), including ABCB1 and ABCG2. Most ABC transport proteins directly contribute to the development of drug resistance, and the enhanced efflux activity of these proteins positively regulates drug resistance (Wang et al., 2018a; Wu et al., 2019). Multidrug resistance (MDR) caused by lung cancer stem cells correlates with the expression of ABCB1 and ABCG2 (Bhukhai et al., 2018; Mohammad et al., 2018; Zhang et al., 2020; Cortes-Dericks and Galetta, 2022). Docetaxel is widely used as a third-generation chemotherapeutic agent for the treatment of patients with NSCLC. However, most patients exhibit drug resistance after a period of treatment. A study of the NSCLC docetaxel-resistant cell lines HCC827-DR found that this resistant cell lines exhibited CSC-like markers and high expression of ABCB1 in all cells (Chen et al., 2017). By pumping chemotherapeutic medications out of cells and lowering the concentration of intracellular pharmaceuticals, ABCG2 significantly increased the chemotherapy resistance of lung cancer stem cells (Huang and Fu, 2015) (Figure 2).

#### 4.1.2 DNA damage repair

CSCs have a higher capacity to repair DNA damage than other types of tumor cells (Masoumi et al., 2021). Ataxia telangiectasia mutated Protein (ATM) and ataxia telangiectasia and Rad3-related (ATR) are members of the phosphatidylinositol 3-kinase (PI3K)-related kinase (PIKK) protein family, and are frequently activated as checkpoint sensors during cancer treatment therapy. When DNA is damaged by chemoradiotherapy, ATM and ATR kinases form complexes with PARP-1 and BRCA1, phosphorylating CHK1 and CHK2 and then promoting the activation of target proteins, including *p53* and *CDC25A*, temporarily halting the cell cycle, thus repairing most DNA damaged by chemoradiotherapy or directly inducing apoptosis (Zhou et al., 2021). In NSCLC, CD133+ lung cancer stem cells were observed to increase the expression of DNA damage response and repair in genes (Desai et al., 2014). Meanwhile, a study demonstrated that lung cancer stem cells could lead to therapy resistance through enhanced DNA repair and reduced uptake of cisplatin (Yu et al., 2017) (Figure 2).

#### 4.1.3 Niche protection

The niche, also known as the tumor microenvironment (TME), is composed of multiple cell types, including tumor cells, endothelial cells, mesenchymal cells, immune cells, and fibroblasts, as well as non-cellular components such as the extracellular matrix (ECM). This niche helps to maintain the stem-like properties of lung cancer stem cells, leading to the development of therapeutic resistance (Plaks et al., 2015; Chiu et al., 2022). Tumor-associated macrophages (TAMs) could induce epithelial-mesenchymal transition (EMT) of cancer cells in NSCLC by activating TGF-β signaling and the *β*-catenin pathway (Zeng et al., 2020). EMT plays a significant role in cancer progression and is associated with the production of cancer stem-like cells (Shibue and Weinberg, 2017). Additionally, the use of EMT markers is a common approach for predicting drug resistance in cancer (Huang et al., 2020). TAMs and cancer-associated fibroblasts (CAFs) from primary NSCLC help generate lung cancer stem cells and maintain their stemness (Chen et al., 2014b). CD10+/GPR77+ CAFs could promote drug resistance in patients with lung cancer by secreting IL-6 and IL-8 to maintain the stemness of cancer stem cells (Su et al., 2018) (Figure 2).

### 4.2 Epigenetic regulation of lung cancer stem cells during therapy resistance

Lung cancer stem cells lead to drug resistance by hijacking the properties of normal stem cells. Therefore, reducing the enrichment of lung cancer stem cells through epigenetic modulation is one way to improve therapeutic efficacy.

Histone deacetylase 1 (HDAC1) is highly expressed in cisplatin-resistant lung cancer cells (Wang et al., 2016). HDAC1, in combination with tribbles pseudokinase 1 (TRIB1), in cisplatin treatment, reduced the activity of oncogene *p53* through its deacetylation and induced the enrichment of lung cancer stem cells. In contrast, the silencing of HDAC1 resulted in reduced expression of the transcription factors, *Nanog* and *Oct4*, in lung cancer stem cells and increased sensitivity to cisplatin treatment (Wang et al., 2017) (Figure 2). In addition, HDAC1 maintains lung cancer cell stemness and induces a drug-resistant phenotype in lung cancer cells by inhibiting miR-200b expression and reducing the targeting of miR-200b to *Suz12* (Chen et al., 2014a). HDAC11 has an effect similar to that of HDAC1, which is highly expressed in the cancer stem cell population of LUAD, resulting in enhanced self-renewal of lung cancer stem cells and interaction with GLI1 to upregulate *SOX2* expression (Zuo et al., 2020). MARCKSL1-2 is a long non-coding RNA that recruits *Suz12* to the promoter of histone deacetylase 1 (HDAC1), increasing the level of H3K27me3 at the HDAC1 promoter, while decreasing HDAC1 expression. Thus, miR-200b expression is upregulated to reverse drug resistance (Jiang et al., 2022).

Although there are many epigenetic targets that have shown advantages in the treatment of lung cancer stem cells, the molecular mechanisms of the upstream and downstream regulation of most targets remain unclear. Additionally, the types of drug resistance that could be improved by these targets are limited.

## 5 Epigenetic regulation in the tumor microenvironment

The tumor microenvironment, especially the tumor immune microenvironment (TIME), is not only related to the resistance of lung cancer treatment, but also influences the whole process of lung cancer development (Ferguson et al., 2021).

The location, type, density, and functional status of immune cells (T cells, B cells, NK cells, DC cells, macrophages, neutrophils, monocytes, and mast cells) within the tumor immune microenvironment contribute to its diversity (Binnewies et al., 2018). Using single-cell technology, significant differences in the immune microenvironments of LUAD and LUSC have been confirmed (Wang et al., 2022). The occurrence, growth, and treatment of tumors are significantly affected by this diversity. Therefore, many studies have focused on the immune landscape of the tumor immune microenvironment. Patients who received neoadjuvant chemotherapy had higher levels of PD-L1 expression and T cell subsets regulation than those who did not receive neoadjuvant chemotherapy, according to a study based on the effects of multiple immunofluorescence and image analysis methods on the immune microenvironment of NSCLC (Parra et al., 2018; Chiu et al., 2022). An analysis of the number, density, and ratio of 26 kinds of immune cells in the tumor immunological microenvironment of 681 NSCLC cases revealed that patients with immunodeficient tumors had shorter disease-free survival and that their tumors had a high number of LCSC and macrophages (Peng et al., 2021). The overall proportion and characteristics of T cells within the TIME are major factors that determine the direction of tumor progression (Mohammad et al., 2018). T cell exhaustion occurs immediately after oncogenic initiation, and some irreversible T cell exhaustion is responsible for the insensitivity of patients to anti-PD-1/ PD-L1 therapy (Pauken et al., 2016; Guo et al., 2018). In the process of T cell exhaustion, inhibitory receptors such as CTLA-4, TIM-3, LAG-3, and PD-1 are usually over-expressed on T cells, and effector cytokines such as IFN-γ are down-regulated (Blank et al., 2019).

**FIGURE 3:**
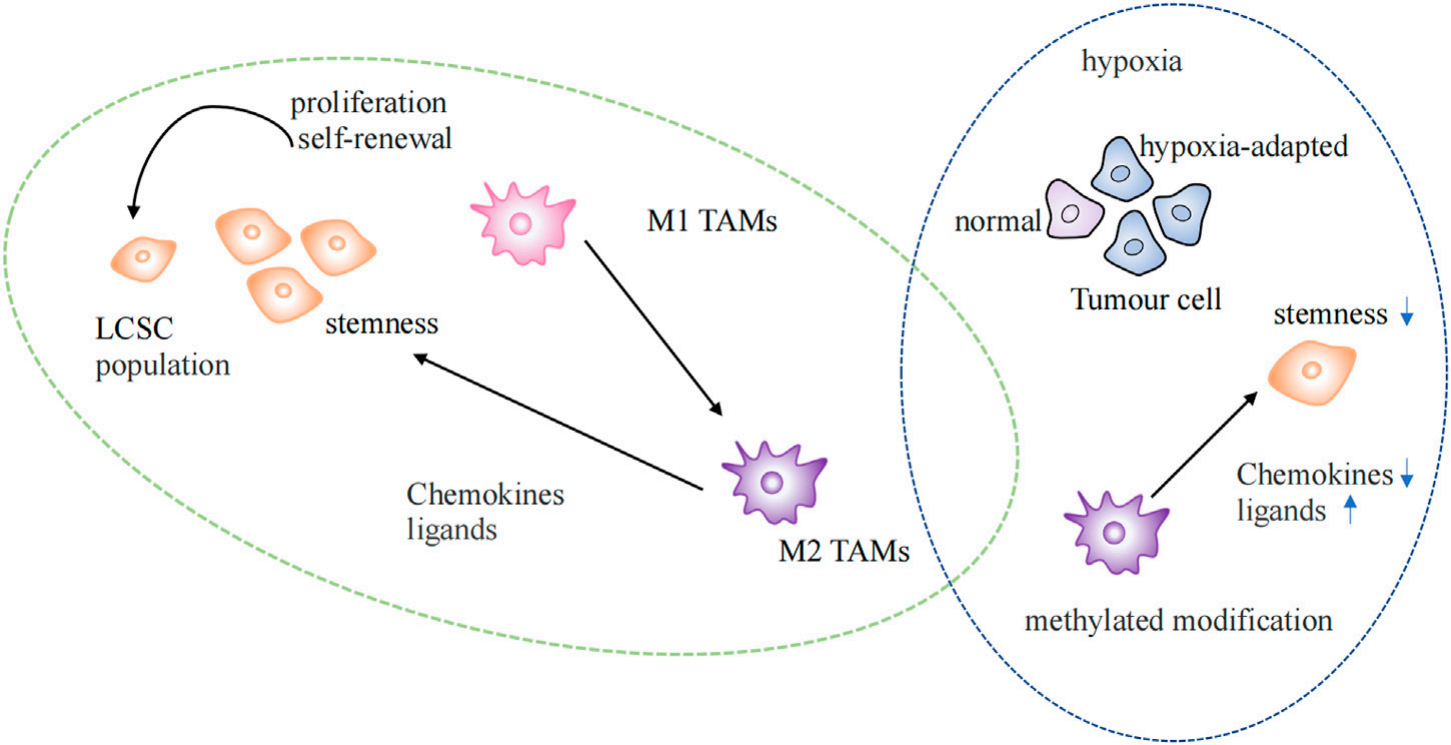
Epigenetic regulation in the tumor microenvironment. Regulation of lung cancer stem cells by TAMs of the M2 phenotype in normal and hypoxic environments. LSCS: lung cancer stem cell; TAMs: Tumor-associated macrophages.

Recent studies have shown that tumor immune microenvironment could be reshaped by epigenetic immune editing (Gangoso et al., 2021). Epigenetic changes could be triggered by inflammation (Karin and Shalapour, 2022). The hypoxia-adapted cellular phenotype is sustained in the tumor microenvironment by the synergistic effect of epigenetic factors and hypoxia-inducible transcription factors (HIF). Extensive DNA methylation and histone modifications occur in the hypoxic TME, promoting tumor growth, increasing invasiveness, and maintaining cancer cell stemness (Wang et al., 2011b; Hu et al., 2021) (Figure 3).

TAMs are currently the most widely studied immunosuppressive cell at the TIME (Mohammad et al., 2018). TAMs gather at the injury site after identifying signals, such as chemokines, cytokines, inflammatory mediators, pathogens, or damage-associated molecular patterns (DAMP), which trigger the inflammatory response. M1 and M2 phenotypes of TAMs exist. The M1 phenotype is characterized by its anticancer activity and typically activated macrophages. After epigenetic reprogramming, M2 phenotype macrophages are formed by differentiation and polarization, which have the potential to promote tumors (Netea et al., 2016). M2 phenotypic TAMs maintain tumor stem cell populations by secreting chemokines and ligands that activate stem cell pathways (Huang et al., 2020) (Figure 3). Enhanced methylation modifications and diminished chemokine expression within TAMs in hypoxic environments alter the immune landscape within the TME (Tausendschon et al., 2011). LARRPM (lncRNA) reduces TET1 binding to the CSF1 promoter in LUAD, resulting in increased DNA methylation of the CSF1 promoter and the inhibition of CSF1 transcription, thereby reducing macrophage M2 polarization and infiltration. At the same time, discovered that negative regulation of TAM contributes to carcinogenesis (Li et al., 2022). The differentiation of T helper (TH) cells are more stable under epigenetic regulation, and the direction of T helper cell differentiation is determined by different histone modification levels at IFNG sites (Karin and Shalapour, 2022). NEAT1 was found highly expressed in lung cancer and interacts with DNA methyltransferase DNMT1 to regulate cytotoxic T cell infiltration in lung cancer by inhibiting the cGAS/STING pathway (Ma et al., 2020). The proliferation, differentiation, and survival of T cells depend on the activity of EZH2 enhancers, which are important epigenetic regulators of gene expression. Notably, GSK126, an EZH2 inhibitor, could alter the TIME, encourage the synthesis of the Th1 chemokines CXCL9 and CXCL10 in tumors, and boost CD8^+^ T cell infiltration (Huang et al., 2019). The presence of tumor-infiltrating B lymphocytes could be observed at all stages of lung cancer development, and it has been found that histone modification could also increase the infiltration of B cells (Wang et al., 2019; Karin and Shalapour, 2022). The epigenetic silencing of NKG2DL in SCLC results in a lack of stimulatory signals to activate NK cells, thereby increasing the aggressiveness and metastasis of SCLC (Zhu et al., 2021).

These studies illustrate that the tumor microenvironment plays an essential role in the progression of lung cancer. In particular, the status of lung cancer stem cells, which is influenced by epigenetic alterations in the tumor microenvironment, is an important cause of treatment resistance and cancer recurrence.

## 6 Epigenetic therapy strategies

Epigenetic-based therapeutic strategies aim to regulate the transcriptional programming of various signaling pathways in immune cells, and cancer cells, thereby affecting the fate of each of these cell populations (Dai et al., 2021) (Table 2). The major epigenetic targets associated with lung cancer treatment are DNA methyltransferase (DNMT), histone lysine methyltransferase (KMT), and histone lysine acetyltransferase. Epigenetic-based drugs are often used in combination with targeted therapies and chemotherapy to enhance their efficacy and reduce drug resistance.

**TABLE 2:** Ongoing clinical trials of epigenetic drugs in lung cancer.

Target	Drug	Combination agent	Tumor type	Trial number
EZH2	PF 06821497	Standard of care	SCLC	NCT03460977
	Tazemetostat	Pembrolizumab	NSCLC	NCT05467748
	Tazemetostat	Topotecan and Pembrolizumab	SCLC	NCT05353439
BET inhibitor	ZEN003694	—	LUSC	NCT05607108
KDM1A	Bomedemstat	Atezolizumab	SCLC	NCT05191797
HDAC	ACY 241	Nivolumab	NSCLC	NCT02635061
	HBI-8000	Pembrolizumab	NSCLC	NCT05141357
	Entinostat	Pembrolizumab	NSCLC	NCT02437136
	Entinostat	Carboplatin and Etoposide	SCLC	NCT04631029
	vorinostat	Pembrolizumab	NSCLC	NCT02638090
	Entinostat + Azacitidine	Nivolumab	NSCLC	NCT01928576
	Vorinostat	Pembrolizumab	LUAD	NCT04357873

SCLC, small-cell lung cancer; NSCLC, non-small cell lung cancer; LUSC, lung squamous cancer; LUAD, lung adenocarcinoma.

Some HDAC and DNMT inhibitors are currently clinically approved, such as the histone deacetylase inhibitors Vorinostat, Romidepsin, Belinostat, and DNA methylation inhibitor diecitabine. Additionally, multiple clinical trials are underway for EZH2 inhibitors, KDM1A inhibitors, and BET proteins (Table 2). Although these drugs currently show a partial advantage, in a study of the efficacy of histone methyltransferase G9a in lung cancer, Rowbotham found that G9a may increase the number of lung cancer stem cells and thus promote lung cancer progression (Rowbotham et al., 2018). In addition, Hypomethylation therapy may lead to demethylation and upregulation of oncogene expression (Liu et al., 2022b). Current studies cannot explain these phenomena; therefore, more in-depth studies on these regulatory mechanisms are warranted in the future.

## 7 Perspectives

As mentioned previously, the stem cell programs control the growth and therapy resistance of lung cancer through epigenetic inheritance. Numerous studies have also shown the significance of WNT, NOTCH, and other traditional stem cell pathways in the onset and progression of lung cancer. Epigenetic modification enzymes, such as DNA methylase, histone deacetylase, and their inhibitory enzymes, play a role in different stem cell pathways. The three main causes of resistance to lung cancer treatment are drug transport, DNA damage repair, and niche protection. ATP-binding cassette transporters greatly increase the chemotherapeutic resistance of lung cancer stem cells by pumping chemotherapeutic medications out of cells and lowering intracellular drug concentrations. ATM and ATR kinases associate with PARP-1 and BRCA1 to form complexes that phosphorylate CHK1 and CHK2, boosting the activation of target proteins and preserving the stemness of LCSC. Through the spatial distribution and composition of different cells, the niche maintains the stemness of lung cancer stem cells. Additionally, epigenetic regulation of the immune microenvironment could also affect the outcome of lung cancer. Although the epigenetic regulation in lung cancer stem cell-related drug resistance regulation is still insufficient compared to genetic regulation, existing research shows great potential.

As expected, the modulation of various cell fates through epigenetic modulators is an effective strategy for lung cancer treatment. However, this type of drug is a double-edged sword. It may also increase the number of lung cancer stem cells, making it difficult for the cancer to be completely eliminated. Therefore, the combination of epigenetic drugs with other drugs or other treatments may be the future direction in lung cancer treatment.
